# Chronic Suppurative Otitis Media in urban private school children of Nepal

**DOI:** 10.1016/S1808-8694(15)30516-4

**Published:** 2015-10-18

**Authors:** Prakash Adhikari, Sambudutta Joshi, Dipal Baral, Binit Kharel

**Affiliations:** 1 Dr. (M.S. Resident., Department of Otorhinolaryngology and Head and Neck Surgery, T. U. Teaching Hospital, Kathmandu, Nepal.); 2 Dr. (Medical Officer, Department of Otorhinolaryngology and Head and Neck Surgery, Himchuli Medical Hall, Kathmandu, Nepal); 3 Dr. (Medical Officer, Department of Neurosurgery, Neuro Hospital, Kathmandu, Nepal.); 4 Dr. (Medical Officer, Department of Otorhinolaryngology and Head and Neck Surgery, Himchuli Medical Hall, Kathmandu, Nepal.) Department of Otorhino-Laryngology and Head and Neck Surgery, T. U. Teaching Hospital, Kathmandu, Nepal

**Keywords:** school children, nepal, chronic suppurative otitis media, prevalence

## Abstract

Chronic suppurative otitis media is one of the common ear diseases of the ear, particularly in childhood. It is the commonest cause of persistent mild to moderate hearing impairment in children and young adults.

**Aim:**

To find out the prevalence of chronic suppurative otitis media among school children studying in urban private schools of Nepal.

**Materials and Methods:**

This study was carried out among 500 school children aged between 5 -15 years. Students were selected from urban private schools of four districts of Nepal. In all students, history was taken and otoscopic examination done from May 2006 to October 2006. Data were described using frequency and percentage. Study design: Prospective cross sectional study.

**Results:**

Results showed that the prevalence of chronic suppurative otitis media in children studying in urban private schools of Nepal is 5.0%. Unilateral disease was seen in 72.0%, 76.0% had a tubotympanic disease and 24.0% had atticoantral disease.

**Conclusion:**

The prevalence of chronic suppurative otitis media in urban private school children in Nepal is higher than other studies done in private school children. Health education, improvement of socioeconomic status and health facilities will be helpful in reducing the prevalence of chronic suppurative otitis media.

## INTRODUCTION

Chronic suppurative otitis media is one of the most common health problems in Nepal. Its incidence has been reported to depend on race and socio-economic factors.^1^ The etiology and pathogenesis of otitis media are multifactorial and include genetic, infections, allergy, environmental, social and racial factors and Eustachian tube dysfunction.^2^ During the recent decades, the incidence of chronic suppurative otitis media has dramatically declined due to improvements in housing, hygiene and antimicrobial chemotherapy.^3^ In the developing countries, there is differential prevalence among the different socioeconomic strata of the community. Okafor found that the majority of the patients with chronic ear disease came from poor communities living in subsistence agricultural or slum areas of the cities.^4^ It is the commonest cause of persistent mild to moderate hearing impairment in children and young adults.^5^ The exact prevalence of CSOM in children of urban private schools is not known. So, this study was done to find out the prevalence of chronic suppurative otitis media (CSOM) among school children studying in urban private schools of Nepal.

## METHODS

This study was conducted from May 2006 to October 2006 in urban private schools of four districts of Nepal. Altogether, there were 500 students aged between 5-15 years. The diagnosis of CSOM was made on history and otoscopic findings. Persistent perforation of the tympanic membrane with or without otorrhoea of more than three months duration was taken as evidence of CSOM. CSOM is usually classified into two main groups: tubotympanic (TT) and atticoantral (AA) disease. Tubotympanic disease is characterized by a perforation of the pars tensa. Atticoantral disease most commonly involves the pars flaccida and is characterized by the formation of a retraction pocket in which keratin accumulates to produce cholesteatoma. Cases of impacted cerumen were given wax dissolving ear drops for 1 week and then dewaxing were done either by syringing or by suctioning the ears. Children of middle class family study in these private schools. The average annual income of the family members of our study group is between Nepalese rupees 1,00,000– 4,00,000. Our study group only includes middle class family children as in Nepal, children of lower class family studies in government schools while upper class family studies outside Nepal or in some selected schools of Nepal. Such schools were excluded from our study. Data were described using frequency and percentage.

## RESULTS

There were 500 students out of which 54.0% were male and 46.0% were female (Table-1). The prevalence of CSOM revealed 5.0 %. Around 76.0% had tubotympanic disease, 24.0% had atticoantral disease and 72.0 % had unilateral CSOM. (Table-2). Around 32.0% were active CSOM (Table-3).

**Table 1:** Distribution of children by sex

Sex	Number of students (Percentage)
Male	270(54.0%)
Female	230 (46.0%)
Total	500 (100.0%)

**Table 2:** Types and side of CSOM

Types of CSOM	No. of students (Percentage)
CSOM-Tubotympanic	
Right	10 (52,7%)
Left	4 (21,0%)
Both	5 (26,3%)
CSOM-Tubotympanic (Total)	19 (76,0%)
CSOM- Atticoantral	
Right	3 (50,0%)
Left	1 (16,7%)
Both	2 (33,3%)
CSOM- Atticoantral (Total)	6 (24,0%)
CSOM (Total)	25 (100,0%)

**Table 3:** Active and Inactive CSOM

CSOM	No. of students (Percentage)
Active	8 (32.0%)
Inactive	17 (68.0%)
Total	25 (100.0%)

## DISCUSSION

Chronic suppurative otitis media (CSOM) is one of the common ear diseases of the ear, particularly in childhood.^6^ Poor living conditions, overcrowding, poor hygiene and nutrition have been suggested as a basis for the widespread prevalence of CSOM in developing countries.^7^ Okafor et al study showed that there were only a few cases where CSOM affected patients from the higher socio-economic ladder and even then the pathology started before the patient moved up the socioeconomic ladder.^4^ The only exceptions to this finding were a few children born in good circumstances but with special problems such as cleft palate.^4^ Researchers agree that a combination of a poor, supportive, unstimulating background and a bearing defect almost, guarantees that a child will not attain his full academic potential.^8^ Unfortunately, the two factors very often co-exist.

CSOM with and without complication continues to affect a large number of patients particularly in developing countries.^9^ Studies in Bangladesh, India, various countries in Africa and among some disadvantaged ethnic groups have shown that CSOM may have a prevalence between 2% and 17 % among children.^10^ In a survey of Nigerian school children aged 6-15 years, a higher rate of tympanic membrane perforations (evidence of CSOM) was found among rural children than in urban children.^11^ The ratio was 4%:1%.^16^

The prevalence of CSOM varies in different countries, different populations and ethnic groups. The prevalence of CSOM in our study was found to be 5.0%. This is similar to the study done by Rupa et al (6%)^12^ and Ologe et al (6%)^9^ in rural communities of south India and Nigeria respectively. However, it is lower than the study done by Biswas et al (12.4%)^5^ and Morris et al study (15.0%). ^13^ The prevalence is lower than the study done by Biswas et al ^5^ and Morris et al ^13^ study because of the fact that our study was done in urban private school children whose socioeconomic status and literacy rate is higher in parents of our study group than that of rural school going children of Bangladesh and young aboriginal children from remote communities in Northern and central Australia. Ologe and Nwawalo et al found the prevalence of CSOM to be 6.0% in rural government primary schools while 0% in the children of urban private schools.^14^ Okeowo et al study observed a statistically significant difference in the prevalence of CSOM among rural school children (3.6%) and urban school children (0.6%).^15^ Our study had a higher prevalence than the private school children of Ologe and Nwawalo et al^14^ study and Okeowo et al^15^ study which might be due to higher in the socioeconomic status in children of their studies. However, the prevalence of CSOM in rural government school children in eastern Nepal as quoted by Maharjan et al study (13.2%)^2^ is similar with the study done by Biswas et al and Morris et al study. Maharjan et al study reflected that the prevalence of CSOM is similar in rural communities of Nepal, Bangladesh and aboriginal children of Northern and central Australia. Probably the habit of swimming in polluted water in a pond or river regularly may be a factor responsible for discharging ear. This habit is more common among rural population in the Terai region of Nepal, especially during summer and monsoon, which also explains why study done by Maharjan et al^8^ had higher prevalence of CSOM as compared to our study which was done in urban private school children. Almost all of our school children (76.0 %) had a tubotympanic type of disease. It correlates with the finding of Ologe et al (99.0%) ^9^. There were only 24.0% children with atticoantral disease. Unilateral diseases is most prevalent in different studies.^3^, ^9^ This recent study also revealed unilateral CSOM (72.0 %) as a common entity. In our study almost all of our school children (88.9%) had a tubotympanic type of diseases which was in between the findings of Kamal et al study (73.4%) 16 and Ologe et al ^9^ (99%). Our study could not find the cases with complication of CSOM, as majority of cases were tubotympanic type, which is a safe disease.

Potential loss of hearing as a result of otitis media has important consequences on the development of speech and cognitive abilities, including academic performance of children.^7^ Thus the gap between the fortunate and the less privileged is further widened by an innate difficulty in learning occasioned by CSOM.^14^ Chronic suppurative otitis media is generally found to be more prevalent in lower socioeconomic condition. Our recent study showed that the CSOM is a common ear disease but lower than in government school children of Nepal. This is due to the study done in the urban private school children where there is a good socioeconomic status, good sanitation, better housing condition and better health care facilities.

## CONCLUSION

The prevalence of chronic suppurative otitis media in urban private school children of Nepal is 5.0% which is lower than other studies done in private school children. The combined effort is necessary to decline its prevalence further. Health education through School health program to prevent ear diseases, which is one of the major causes of deafness, is in need to reduce the prevalence of chronic suppurative otitis media and prevent the school children from disability.
